# Association between osteoarthritis and unmet medical needs in Korea: limitations in activities as a mediator

**DOI:** 10.1186/s12889-020-09140-3

**Published:** 2020-06-29

**Authors:** Hooin Jo, Eun-san Kim, Boyoung Jung, Soo-Hyun Sung, In-Hyuk Ha

**Affiliations:** 1 Jaseng Hospital of Korean Medicine, Gangnam-daero, Gangnam-gu, Seoul, Republic of Korea; 2 Jaseng Spine and Joint Research Institute, Jaseng Medical Foundation, Gangnam-daero, Gangnam-gu, Seoul, Republic of Korea; 3 Department of Health Administration, Hanyang Women’s University, Salgoji-gil, Seongdong-gu, Seoul, Republic of Korea; 4 National Development Institute of Korean Medicine, Toegye-ro, Jung-gu, Seoul, Republic of Korea

**Keywords:** Osteoarthritis, Limitations in activities, Unmet medical needs, Mediation analysis

## Abstract

**Background:**

The role of limitations in activities in relation to unmet needs is not clarified. This study aimed to analyze the effects of osteoarthritis on unmet medical needs and the mediating effects of limitations in activities.

**Methods:**

A total number of 10,129 population aged ≥50 years were included using data from the Korean National Health and Nutrition Examination Survey from January 2010 to December 2013. Osteoarthritis was defined as Kellgren-Lawrence grade ≥ 2 in the knee, hip, and lumbar spine joints with pain reported to have lasted for ≥3 months. Limitations in activities were defined as currently experiencing restricted daily and social activities. Unmet medical needs were analyzed after they were further divided into availability, accessibility, and acceptability. Causal mediation analysis was employed to analyze mediating effects.

**Results:**

The osteoarthritis group had a higher odds ratio (OR: 1.65; 95% confidence interval [CI], 1.56–1.75) for the total effects of osteoarthritis on unmet medical needs than the non-osteoarthritis group. Furthermore, the OR for the indirect effects mediated by limitations in activities was higher in the osteoarthritis group (OR: 1.07; 95% CI, 1.05–1.08), indicating that 13.2% of the total effect was mediated. When the analysis was further classified according to cause, the mediating effect of limitations in activities was the strongest at 23.9% for unmet medical needs due to lack of transportation accessibility.

**Conclusions:**

Osteoarthritis exerts significant effects on the experience of unmet medical needs, and limitations in activities mediate such experiences of unmet medical needs in osteoarthritis patients.

## Background

Osteoarthritis involves the loss of normal skeletal structure, cartilage damage, and ligament stiffness as osteoblast differentiation declines and as cartilage undergoes more frequent degradation with aging [1]. Osteoarthritis is one of the most common causes of disability and induces pain, stiffness, edema, and functional decline in the affected joint, leading to limitations in activities [2]. According to the definition by the World Health Organization, osteoarthritis can result in not only difficulty in performing daily activities but also physical and psychological problems such as depression, feeling of helplessness, and sense of alienation caused by pain and functional disability [3]^.^

Korea became an aging society in 2000, and the population aged 65 years or older accounted for more than 14% in 2017 and 15.1% in 2019 [4]. The prevalence of osteoarthritis is likely to consistently increase because its prevalence increases with aging, which further increases the number of osteoarthritis patients [5]. According to the National Health Insurance Service, there were 4,082,690 osteoarthritis patients in 2011, which increased to 4,491,909 osteoarthritis patients in 2015, indicating a 10% increase.

A healthcare needs is defined as “required medical services that can appropriately prevent, alleviate, and cure the status of illness or inability caused by some disturbance in health and well-being” [6]. In terms of health policy, unmet needs are identified with respect to three aspects—availability, accessibility, and acceptability [7]. First, availability includes cases when medical service is not available due to long waiting time or lack of medical resources in certain residential areas. Second, accessibility has the strongest association with medical social security. In a society where medical social security is not well established, financial ability that allows access to medical service is the most important factor for unmet medical needs. Transportation service also falls under this category, which can be a big problem for older patients and patients with disability who have difficulty in mobility. Third, acceptability is solely dependent on patients even when medical service is available and accessible. This includes cases in which patients are not provided with medical service as they ignore their own health problems, do not know where to visit, or feel scared or doubtful about doctors.

It is well known that osteoarthritis limits activities [8]. Further, limitation of activities is a known risk factor for unmet medical needs [9]. In the past, studies on the unmet needs among patients with osteoarthritis have examined the clinical aspect only, with no study investigating the role of limitation of activities and consequently perceived unmet needs among patients [10, 11]. Thus, this study aimed to analyze the effects of osteoarthritis on unmet medical needs and the mediating effects of limitations in activities.

## Methods

### Study design and population

This study used data from the 5th and 6th Korean National Health and Nutrition Examination Survey (KNHANES) from January 2010 to December 2013. The goal of KNHANES is to create a statistical database of the national state of health, health behavior, and food and nutritional intake with reliability and representativeness for cities, provinces, and the nation. The study population consisted of citizens residing in Korea (excluding foreigners and those residing in nursing homes, military bases, and prisons). To improve the representativeness of samples and the accuracy of estimation, enumeration districts were extracted after classifying residence and housing type, followed by sampling design.

**Fig. 1:**
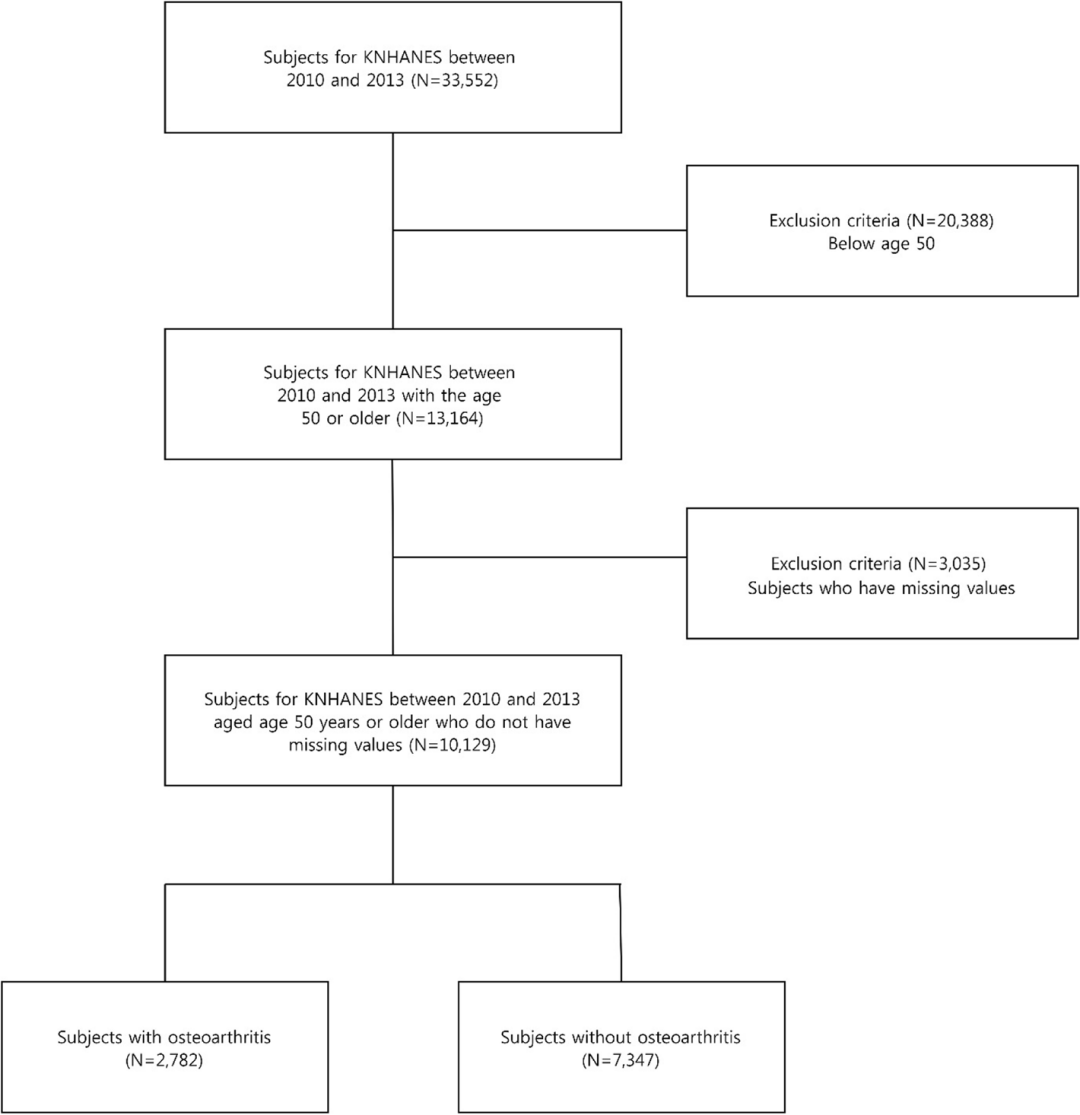
Flowchart of the selected study population

To determine the prevalence of osteoarthritis, KNHANES data from 2010 to 2013, which include osteoarthritis examination data, were used. In 2010 and 2013 KNHANES, a total of 33,552 and 13,164 subjects aged 50 years or older underwent radiographic examination for osteoarthritis, respectively. Moreover, 10,129 subjects did not have missing covariate values. Among them, 8264 patients had Kellgren-Lawrence (KL) grade ≥ 2. Finally, 2782 patients were diagnosed with osteoarthritis (27.5%). The number was adjusted to 4782 after conducting propensity score matching (PSM) for sensitivity analysis. The exclusion criteria for study subjects are presented in Fig. 1.

### Exposure, mediator and outcome

#### Osteoarthritis

Osteoarthritis was defined as KL grade ≥ 2 in the knee, hip, and lumbar spine joints with pain reported to have lasted for ≥3 months, according to the criteria by the Korea Centers for Disease Control and Prevention [12]. For radiographic diagnosis, the knee, hip, and lumbar spine joints were evaluated by radiography. Osteoarthritis grade was determined by two radiology specialists. For the final decision of the radiographic grade, one examiner assessed the results from two examiners. When the grades from these two examiners were inconsistent, the examiner assessing the results chose a higher grade. When the results from the two examiners were different by ≥2 grades, another professor in radiology was asked to re-evaluate the result as a 3rd person. When ≥2 results were identical from the results by the three examiners, their grade was chosen. When all three results were different, the grade determined by the 1st examiner was chosen [13]. To better reflect the actual effects of osteoarthritis, lumbar osteoarthritis was diagnosed in subjects aged 50 years or older who experienced lumbar pain within the past 3 months for ≥30 days and showed KL grade ≥ 2 in radiography [14, 15].

#### Unmet medical needs

In this study, unmet medical needs were assessed based on self-reported unmet healthcare needs. The experience of unmet medical needs was determined from the question “Have you been unable to go to hospital/clinic (excluding dental clinic) when you wanted to within the past year?” in the 5th and 6th KNHANES. To those who responded “yes,” another question “If so, what was the reason you could not go to hospital/clinic when you wanted to among the following?” was asked. The reasons consisted of availability (“The hospital/clinic was not open when I could visit,” “I did not want to wait for a long time at the hospital/clinic”), acceptability (“It was difficult to make a reservation at the hospital/clinic,” “The symptoms were mild”), and accessibility (“Transportation system was inconvenient,” “Due to financial reasons”) [7]. In this study, the overall unmet medical needs were first analyzed as an outcome, and analysis was conducted by setting the incidence of unmet medical needs caused by a specific reason as an outcome. As mediating effects of limitations in activities were analyzed in this study, accessibility was further divided into sub-items.

#### Limitations in activities

The subjects for a health interview on limitations in activities were 1 year or older than the subjects in KNHANES. In this study, the status of limitations in activities was adopted to differentiate subjects with and without limitations in activities. Subjects who responded “yes” to a question, “Are you experiencing limitations in daily and social activities due to health problems or physical/psychological disability?” were classified as having limitations in activities.

### Confounders

#### Sociodemographic factors

Age is major risk factor in OA and limitation in activities [16]., Previous studies showed that the prevalence of osteoarthritis and percentage of experiencing unmet medical needs is different between men and women [17–19]. Other adjusted socioeconomic covariates include Marriage status [14, 19], Household composition [14, 19], Household income(4 quantile) [14, 20, 21], Residence, Education level [14, 17, 22], Occupation [23], Type of insurance [17], and Private insurance [24].

#### Disease factors

Obesity has association with both osteoarthritis [23] and limitations in activities [25]. Obesity was diagnosed by body weight measurement. After zero was indicated on the scale, weight was measured after the number became stabilized, with a unit of 100 g. Obesity was defined as body mass index (kg/m^2^) of 25 kg/m^2^ or higher.

The classification of hypertension, diabetes, and hyperlipidemia followed the standards by the Korea Centers for Disease Control and Prevention. Hypertension was diagnosed as 1) systolic blood pressure of ≥140 mmHg, 2) diastolic blood pressure of ≥90 mmHg, or 3) when the patient was taking antihypertensives. Blood pressure was measured using a mercury sphygmomanometer. The brachial artery of the right arm was palpated, and the middle part of the rubber cuff was placed on top. After the 1st blood pressure reading, the same process was repeated for the 2nd and 3rd blood pressure readings. The mean value of the 2nd and 3rd blood pressure readings was calculated and used to diagnose hypertension [26].

Diabetes and hyperlipidemia were diagnosed through a blood test. The blood sugar level was measured to diagnose diabetes. Among the subjects who fasted for ≥8 h, diabetes was diagnosed when the fasting blood sugar level was ≥126 mg/dL, the diagnosis was made by a doctor, the subject was taking a hypoglycemic agent, or the subject was administering insulin injection. Hypercholesterolemia was diagnosed by measuring the total cholesterol level. Among the subjects who fasted for ≥8 h, hypercholesterolemia was diagnosed when the total cholesterol level was ≥240 mg/dL or when the subject was taking a cholesterol-lowering drug.

Depression is associated with pain caused by osteoarthritis, which is one of the types of chronic pain [27, 28]. To diagnose depression, the subjects were asked whether they were “diagnosed by a doctor” or “currently suffering from depression” through a questionnaire, and the subjects who responded “yes” to both questions were diagnosed with depression.

#### Lifestyle

Muscle weakness is major factor of limitation in activities [2]. This can be prevented with appropriate exercise [29]. The criteria on lifestyle were based on the major indices of the health survey from KNHANES [12]. For the execution of muscle strengthening exercise, subjects who responded that they executed muscle strengthening exercise such as push-up, sit-up, dumbbell, weightlifting, and iron bar for ≥2 days in the past week were categorized as the execution group. The current smoking rate was defined as a percentage of subjects aged 19 years or older who had smoked five packs of cigarettes (100 cigarettes) throughout their life and were also currently smoking. Monthly drinking rate was defined as a percentage of subjects aged 12 years or older who drank once or more per month in the past year.

### Statistical analysis

**Fig. 2:**
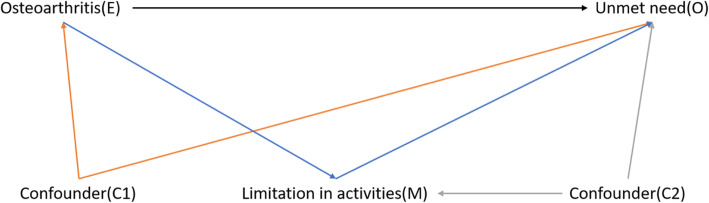
Direct acyclic graph (DAG) showing the causal pathway of osteoarthritis effects and mediating effects of limitations in activities on unmet needs. Illustrative directed acyclic graph of the causal effect on unmet need. *E* exposure, *O* outcome, *M* mediator, *C1* confounder in the natural direct effect, *C2* confounder in the natural indirect effect

The causal diagram of the study is presented in Fig. 2. Using causal mediation analysis (CMA), we estimated the total effect (TE) and natural direct effect (NDE) of osteoarthritis as well as the natural indirect effect (NIE) of limitations in activities. Causal estimate could not be determined from a nonlinear model (e.g., logistic regression) using conventional mediation analysis; hence, CMA, which has been used in public health policy and epidemiological studies [30–32], was thus used to overcome such limitation. All analyses were performed using mediation package in R studio.

In CMA, meeting the sequential ignorability assumption (SIA) is key. To meet the SIA, [1] osteoarthritis must be independent of unmet medical needs and limitations in activities and [2] limitations in activities must be independent of unmet medical needs [33]. To meet such assumption in a cross-sectional study, covariates were controlled via comprehensive literature review in this study.

In estimation, quasi-Bayesian Monte Carlo was conducted for 1000 times in total, and all estimates were converted to odds ratios (OR). The study by VanderWeele et al. was referenced in the process of OR conversion [34]. Additionally, the proportion of NIE in TE was estimated, and interaction analysis was conducted according to limitations in activities.

To meet the SIA, PSM was conducted for sensitivity analysis. For PSM, the probability for the prevalence of osteoarthritis was estimated through covariates using the nearest method. Although 0.2 is used as a proper caliper in the nearest method [35], 0.01 was used instead because covariate balance could not be achieved using a caliper of 0.2 in our study (Additional file 1).

## Results

Table 1 summarizes the general characteristics of the study population and the experience of unmet medical needs according to the prevalence of osteoarthritis. The experience of unmet medical needs showed a higher percentage in the osteoarthritis group. Among sociodemographic factors, the mean age was higher in the osteoarthritis group (67.1 ± 8.6) than in the non-osteoarthritis group (61.7 ± 8.4), and the percentage of women (73.3%, *n* = 2040) was higher than the percentage of men (26.7%, *n* = 742) in the osteoarthritis group. Socioeconomic status was lower and the prevalence of chronic disease was higher in the osteoarthritis group than in the non-osteoarthritis group.

**Table 1:** Sociodemographic characteristics of the population

	OA	Non-OA	
	**(*N*** **= 2782)**	**(*N*** **= 7347)**	***P*value**
**Age (years)**	67.1 ± 8.6	61.7 ± 8.4	< 0.001
**Sex**			< 0.001
Men	742 (26.7)	3638 (49.5)	
Women	2040 (73.3)	3709 (50.5)	
**Marriage status**			< 0.001
Cohabitant, spouse	1883 (67.7)	6168 (84.0)	
Divorced, separated, single, widowed	899 (32.3)	1179 (16.0)	
**Household composition**			< 0.001
One-person household	495 (17.8)	621 (8.5)	
One-generation household	1117 (40.2)	2702 (36.8)	
Two-generation household	818 (29.4)	3233 (44.0)	
Three-generation household or more	352 (12.7)	791 (10.8)	
**Household income**			< 0.001
Lower	1264 (45.4)	1718 (23.4)	
Lower middle	719 (25.8)	1908 (26.0)	
Upper middle	429 (15.4)	1757 (23.9)	
Upper	370 (13.3)	1964 (26.7)	
**Residence**			< 0.001
Town	1756 (63.1)	5694 (77.5)	
Rural	1026 (36.9)	1653 (22.5)	
**Education level**			< 0.001
Elementary school graduate or lower	1906 (68.5)	2687 (36.6)	
Middle school graduate	416 (15.0)	1350 (18.4)	
High school graduate	344 (12.4)	2176 (29.6)	
College graduate or higher	116 (4.2)	1134 (15.4)	
**Occupation**			< 0.001
White collar and service	1554 (55.9)	3178 (43.3)	
Blue collar and farmer	294 (10.6)	1655 (22.5)	
Unemployed	934 (33.6)	2514 (34.2)	
**Type of insurance**			< 0.001
Medical aid system	148 (5.3)	183 (2.5)	
National health insurance	2634 (94.7)	7164 (97.5)	
**Private insurance**			< 0.001
No	1555 (55.9)	2610 (35.5)	
Yes	1227 (44.1)	4737 (64.5)	
**Current status of smoking**			< 0.001
No	2462 (88.5)	6098 (83.0)	
Yes	320 (11.5)	1249 (17.0)	
**Monthly status of drinking**			< 0.001
No	1854 (66.6)	3795 (51.7)	
Yes	928 (33.4)	3552 (48.3)	
**Execution of muscle strengthening exercise**			< 0.001
No	2445 (87.9)	5567 (75.8)	
Yes	337 (12.1)	1780 (24.2)	
**Depression**			< 0.001
No	2658 (95.5)	7185 (97.8)	
Yes	124 (4.5)	162 (2.2)	
**Obesity**			< 0.001
No	1638 (58.9)	4925 (67.0)	
Yes	1144 (41.1)	2422 (33.0)	
**Hypertension**			< 0.001
No	1155 (41.5)	3927 (53.5)	
Yes	1627 (58.5)	3420 (46.5)	
**Diabetes**			< 0.001
No	2230 (80.2)	6119 (83.3)	
Yes	552 (19.8)	1228 (16.7)	
**Hyperlipidemia**			< 0.001
No	2072 (74.5)	5759 (78.4)	
Yes	710 (25.5)	1588 (21.6)	

Values are presented as number (percentage) and mean ± standard deviation. *OA:* osteoarthritis;

Table 2 shows the general characteristics of the study population according to osteoarthritis and limitations in activities. In the osteoarthritis group, the percentage of unmet medical needs was higher in subjects with limitations in activities than in subjects without limitations in activities. When unmet medical needs were divided into availability, accessibility, and acceptability according to cause, the percentage of unmet medical needs due to accessibility was higher in subjects with limitations in activities than in subjects without limitations in activities. When unmet medical needs due to accessibility were divided into financial accessibility and transportation accessibility, the trend was consistent. In the non-osteoarthritis group, the percentage of unmet medical needs was higher in the group with limitations in activities. However, according to limitations in activities, no significant difference in unmet medical needs due to availability was found in both the osteoarthritis and non-osteoarthritis groups.

**Table 2:** General characteristics of subjects by mediators

	OA (***N*** = 2782)			Non-OA (***N*** = 7347)		
**Limitations in activities**	**Yes**	**No**	***P*value**	**Yes**	**No**	***P*value**
	(*N* = 877)	(*N* = 1905)		(*N* = 657)	(*N* = 6690)	
**Age (years)**	68.5 ± 8.4	66.4 ± 8.6	< 0.001	63.7 ± 8.5	61.5 ± 8.4	< 0.001
**Sex**			0.923			0.057
Men	255 (29.1)	487 (25.6)		327 (49.8)	3311 (49.5)	
Women	622 (70.9)	1418 (74.4)		330 (50.2)	3379 (50.5)	
**Marriage status**			< 0.001			0.007
Cohabitant, spouse	562 (64.1)	1321 (69.3)		508 (77.3)	5660 (84.6)	
Divorced, separated, single, widowed	315 (35.9)	584 (30.7)		149 (22.7)	1030 (15.4)	
**Household composition**			< 0.001			0.001
One-person household	190 (21.7)	305 (16.0)		82 (12.5)	539 (8.1)	
One-generation household	354 (40.4)	763 (40.1)		273 (41.6)	2429 (36.3)	
Two-generation household	233 (26.6)	585 (30.7)		238 (36.2)	2995 (44.8)	
Three-generation household or more	100 (11.4)	252 (13.2)		64 (9.7)	727 (10.9)	
**Household income**			< 0.001			< 0.001
Lower	477 (54.4)	787 (41.3)		247 (37.6)	1471 (22.0)	
Lower middle	219 (25.0)	500 (26.2)		176 (26.8)	1732 (25.9)	
Upper middle	107 (12.2)	322 (16.9)		135 (20.5)	1622 (24.2)	
Upper	74 (8.4)	296 (15.5)		99 (15.1)	1865 (27.9)	
**Residence**			0.034			0.007
Town	521 (59.4)	1235 (64.8)		487 (74.1)	5207 (77.8)	
Rural	356 (40.6)	670 (35.2)		170 (25.9)	1483 (22.2)	
**Education level**			< 0.001			< 0.001
Elementary school graduate or lower	645 (73.5)	1261 (66.2)		319 (48.6)	2368 (35.4)	
Middle school graduate	133 (15.2)	283 (14.9)		118 (18.0)	1232 (18.4)	
High school graduate	76 (8.7)	268 (14.1)		151 (23.0)	2025 (30.3)	
College graduate or higher	23 (2.6)	93 (4.9)		69 (10.5)	1065 (15.9)	
**Occupation**			< 0.001			< 0.001
White collar and service	49 (5.6)	245 (12.9)		78 (11.9)	1577 (23.6)	
Blue collar and farmer	239 (27.3)	695 (36.5)		172 (26.2)	2342 (35.0)	
Unemployed	589 (67.2)	965 (50.7)		407 (61.9)	2771 (41.4)	
**Type of insurance**			< 0.001			< 0.001
Medical aid system	78 (8.9)	70 (3.7)		61 (9.3)	122 (1.8)	
National health insurance	799 (91.1)	1835 (96.3)		596 (90.7)	6568 (98.2)	
**Private insurance**			< 0.001			< 0.001
No	548 (62.5)	1007 (52.9)		345 (52.5)	2265 (33.9)	
Yes	329 (37.5)	898 (47.1)		312 (47.5)	4425 (66.1)	
**Current status of smoking**			0.223			0.106
No	763 (87.0)	1699 (89.2)		557 (84.8)	5541 (82.8)	
Yes	114 (13.0)	206 (10.8)		100 (15.2)	1149 (17.2)	
**Monthly status of drinking**			< 0.001			0.015
No	613 (69.9)	1241 (65.1)		409 (62.3)	3386 (50.6)	
Yes	264 (30.1)	664 (34.9)		248 (37.7)	3304 (49.4)	
**Execution of muscle strengthening exercise**			0.024			0.01
No	792 (90.3)	1653 (86.8)		522 (79.5)	5045 (75.4)	
Yes	85 (9.7)	252 (13.2)		135 (20.5)	1645 (24.6)	
**Depression**			< 0.001			< 0.001
No	807 (92.0)	1851 (97.2)		610 (92.8)	6575 (98.3)	
Yes	70 (8.0)	54 (2.8)		47 (7.2)	115 (1.7)	
**Obesity**			0.607			0.401
No	527 (60.1)	1111 (58.3)		434 (66.1)	4491 (67.1)	
Yes	350 (39.9)	794 (41.7)		223 (33.9)	2199 (32.9)	
**Hypertension**			0.052			0.034
No	338 (38.5)	817 (42.9)		327 (49.8)	3600 (53.8)	
Yes	539 (61.5)	1088 (57.1)		330 (50.2)	3090 (46.2)	
**Diabetes**			< 0.001			0.113
No	687 (78.3)	1543 (81.0)		501 (76.3)	5618 (84.0)	
Yes	190 (21.7)	362 (19.0)		156 (23.7)	1072 (16.0)	
**Hyperlipidemia**			0.003			0.417
No	644 (73.4)	1428 (75.0)		485 (73.8)	5274 (78.8)	
Yes	233 (26.6)	477 (25.0)		172 (26.2)	1416 (21.2)	
Unmet needs						
**Unmet needs in all causes**	365 (41.6)	513 (26.9)	< 0.001	141 (21.5)	750 (11.2)	< 0.001
**Availability**	38 (4.3)	133 (7.0)	0.009	31 (4.7)	220 (3.3)	0.07
**Accessibility**	206 (23.5)	205 (10.8)	< 0.001	65 (9.9)	181 (2.7)	< 0.001
**Financial accessibility**	165 (18.8)	175 (9.2)	< 0.001	57 (8.7)	157 (2.3)	< 0.001
**Transportation accessibility**	41 (4.7)	30 (1.6)	< 0.001	8 (1.2)	24 (0.4)	0.004
**Acceptability**	60 (6.8)	126 (6.6)	0.888	30 (4.6)	292 (4.4)	0.888

Values are presented as number (percentage) and mean ± standard deviation. *OA:* osteoarthritis;

Table 3 shows the effects of osteoarthritis on unmet medical needs using limitations in activities as a mediating variable. The OR for the TE of osteoarthritis on unmet medical needs was 1.65 (95% confidence interval [CI], 1.56–1.75). The OR for the NDE was 1.54 (95% CI, 1.46–1.65), and the OR for the NIE mediated by limitations in activities was 1.07 (95% CI, 1.05–1.08), indicating that 13.2% of TE was mediated. When unmet medical needs were divided according to cause, the OR for the TE on accessibility was 2.25 (95% CI, 1.97–2.57). The OR for the NDE was 1.94 (95% CI, 1.69–2.25), and the OR for the NIE mediated by limitations in activities was 1.16 (95% CI, 1.11–1.20). Hence, 18.4% of TE was mediated, showing the strongest mediating effects. Unmet medical needs due to accessibility were divided into financial and transportation for the analysis. The OR for TE resulting from financial accessibility was 2.31 (95% CI, 1.97–2.68). The OR for NDE was 2.01 (95% CI, 1.70–2.34), and the OR for NIE mediated by limitations in activities was 1.15 (95% CI, 1.11–1.20), showing that 17.5% of TE was mediated. The OR for TE due to transportation accessibility was 2.43 (95% CI, 1.57–3.61). The OR for NDE was 1.99 (95% CI, 1.30–3.00), and the OR for NIE mediated by limitations in activities was 1.22 (95% CI, 1.10–1.37), showing that 23.9% of TE was mediated. Among accessibility factors, limitations in activities had the strongest mediating effect on transportation.

**Table 3:** Total effects, natural direct effects and natural indirect effects of osteoarthritis and limitations in activities on unmet needs

	TE	NDE	NIE	% of total effect mediated	Interaction (***P*** value)
**All causes of unmet needs**	1.65 (1.56–1.75)	1.54 (1.46–1.65)	1.07 (1.05–1.08)	13.2	0.11
**Availability**	2.22 (1.83–2.65)	2.13 (1.73–2.57)	1.04 (1.01–1.08)	5.4	0.03
**Accessibility**	2.25 (1.97–2.57)	1.94 (1.69–2.25)	1.16 (1.11–1.20)	18.4	0.21
**Financial accessibility**	2.31 (1.97–2.68)	2.01 (1.70–2.34)	1.15 (1.11–1.20)	17.5	0.54
**Transportation accessibility**	2.43 (1.57–3.61)	1.99 (1.30–3.00)	1.22 (1.10–1.37)	23.9	0.25
**Acceptability**	1.35 (1.13–1.63)	1.34 (1.11–1.62)	1.01 (0.97–1.05)	3.5	0.92

Effects on unmet needs are presented as odds ratio (95% confidence interval). *TE* total effects, *NDE* natural direct effects, *NIE* natural indirect effects

The distribution of subgroup according to the severity is presented in Additional files 1 and 2. The OR for the TE of multi joint OA on unmet medical needs was 1.61 (95% CI, 1.53–1.68), which was higher than OR of single join OA (OR: 1.45; 95% CI, 1.38–1.54). In multi joint OA, the mediating proportion of TE was 11.8%, which was lower than single joint OA (14.4%). However, in the unmet need with transportation, the proportion slightly increased from 23.4 to 23.7% (Table 4). In sensitivity analysis with propensity score matching, the trend of main analysis was consistent (Additional files 3 and 4).

**Table 4:** Subgroup analysis according to severity in osteoarthritis

Mediator variables	Total Effect	Natural Direct Effect	Natural Indirect Effect	% of total effect mediated	Interaction(***P*** value)
**Single joint OA**					
**All cause of unmet need**	1.45 (1.38–1.54)	1.38 (1.31–1.46)	1.05 (1.04–1.07)	14.4	0.16
**Availability**	2.00 (1.66–2.39)	1.92 (1.59–2.31)	1.04 (1.01–1.08)	5.8	0.29
**Accessibility**	1.86 (1.65–2.08)	1.65 (1.46–1.86)	1.13 (1.09–1.16)	19.5	0.17
**financial**	1.93 (1.67–2.21)	1.72 (1.49–1.97)	1.12 (1.08–1.17)	18.2	0.19
**transportation**	2.10 (1.35–3.01)	1.77 (1.12–2.61)	1.19 (1.08–1.31)	23.4	0.45
**Acceptability**	1.29 (1.06–1.55)	1.28 (1.05–1.55)	1.01 (0.97–1.04)	2.5	0.53
**Multi joint OA**					
**All cause of unmet need**	1.61 (1.53–1.68)	1.52 (1.45–1.59)	1.06 (1.04–1.08)	11.8	0.81
**Availability**	2.64 (2.10–3.22)	2.50 (1.95–3.10)	1.06 (1.01–1.12)	6.1	0.10
**Accessibility**	2.59 (2.28–2.94)	2.21 (1.93–2.52)	1.17 (1.12–1.23)	17.5	0.32
**financial**	2.74 (2.37–3.15)	2.33 (2.00–2.69)	1.18 (1.12–1.24)	16.7	0.78
**transportation**	3.04 (1.90–4.63)	2.38 (1.44–3.58)	1.28 (1.13–1.46)	23.7	0.20
**Acceptability**	1.54 (1.14–1.96)	1.52 (1.11–1.99)	1.01 (0.96–1.07)	3.3	0.42

The control group is non-OA population. Effects on unmet needs are presented as odds ratio (95% confidence interval). *TE* total effects, *NDE* natural direct effects, *NIE* natural indirect effects

## Discussion

An unmet medical need is an index reflecting an issue of accessibility to medical service and is therefore critical from a political perspective [36]. Our study showed that osteoarthritis leads to unmet medical needs, and it has the strongest effects on unmet medical needs due to accessibility. The mediation by limitations in activities was also the strongest in this category. Osteoarthritis is characterized by pain, stiffness, and consequent gait abnormality and limitations in activities [37]. This makes transportation accessibility to medical resources difficult, which is supported by a strong mediating effect of limitations in activities on unmet medical needs. Moreover, the proportion of mediating effect on unmet needs due to the transportation was higher among patients with multi-joint OA than those with single-joint OA. From a political aspect, this proposes that transportation accessibility should be improved for osteoarthritis patients by offering transportation options for either home care visit or hospital visit.

Osteoarthritis patients also suffer from the huge burden of medical expenses [38]. Osteoarthritis is known to increase catastrophic health expenditure and have higher average amount of payment than other chronic diseases (e.g., hypertension, hyperlipidemia, diabetes) [39]. Previous studies have shown that a direct cost such as treatment fee and an indirect cost such as travel cost are also the cause of unmet medical needs [40], and our study results also support this finding (OR for TE on unmet medical needs due to financial accessibility: 2.31; 95% CI, 1.97–2.68). Furthermore, our results showed that limitations in activities mediate unmet medical needs due to financial accessibility in osteoarthritis patients by 17.5%. Limitations in activities imply limitations not only in daily activities but also in social activities. The patient group with limitations in activities in our study had overall low socioeconomic status, which indicates that financial aid is needed for osteoarthritis patients.

The mediating effects of limitations in activities were not as strong as in availability and acceptability. When accessibility is also taken into account, this can be interpreted that there are local hospitals and clinics for osteoarthritis patients, but they do not have access to visit these facilities. The exact cause for this matter should be determined in further qualitative studies. Further, the proportion of the mediating effect on all cause of unmet need decreased in patients with multi-joint OA. This highlights the need for studies on the mediating effects other than limitation of activities among OA patients with severe symptoms.

This study has a few limitations. First, there is a possibility of reverse-causality, where OA occurs from the failure to receive proper health care. However, unmet medical needs in this study was defined as any experience of unmet healthcare needs in the past year and OA is a disease that results from an accumulation of several factors for a prolonged period. Although prolonged unmet medical needs may have had an impact on an accumulation, this effect could not be analyzed with a cross-sectional design. Second, although covariates were adjusted through as much literature review as possible, some of the covariates might have been caused by osteoarthritis rather than causing osteoarthritis. Due to this limitation, we performed PSM to reduce the confounding effect, but it was only performed on independent variables, and not on the mediators. These limitations should be complemented by instrumental variables or prospective studies. Finally, this study defined unmet medical needs based on subjective experiences. This is vulnerable to a recall bias and cannot encompass the entire definition of unmet needs. This should be complemented in subsequent studies by using comprehensive indices to examine unmet needs among OA patients.

## Conclusions

By analyzing the data from 2010 to 2013 KNHANES, our study showed that osteoarthritis exerts significant effects on unmet medical needs among Korean adults and that limitations in activities mediate such effects. The mediating effects were particularly increased for unmet medical needs due to transportation accessibility and financial accessibility. Such findings can be utilized in policy making to improve the accessibility of osteoarthritis patients to medical needs. Detailed analysis through further additional studies is required to determine the cause of unmet medical needs in osteoarthritis patients.

## Supplementary information

**Additional file 1.** Table S1. Sociodemographic characteristics of the population according to severity. Values are presented as number (percentage) and mean ± standard deviation. OA: Osteoarthritis.

**Additional file 2.** Table S2. Sociodemographic characteristics of the population according to severity by mediators. Values are presented as number (percentage) and mean ± standard deviation. OA: Osteoarthritis.

**Additional file 3.** Figure S1. Propensity score distribution in the overall and matched study populations. 0.01 caliper was used for matching.

**Additional file 4.** Table S3. Effects on unmet needs are presented as odds ratio (95% confidence interval). Propensity score matching using nearest method with 0.01 caliper was used. TE total effects, NDE natural direct effects, NIE natural indirect effects.

## Data Availability

The datasets used and analyzed during the current study are available from the corresponding author on reasonable request. KNHANES data can be accessed and downloaded from the KNHANES website (https://knhanes.cdc.go.kr/knhanes/index.do).

## Abbreviations

CI: Confidence interval
CMA: Causal mediation analysis
KL: Kellgren-Lawrence
KNHANES: Korean National Health and Nutrition Examination Survey
NDE: Natural direct effect
NIE: Natural indirect effect
OR: Odds ratio
PSM: Propensity score matching
SIA: Sequential ignorability assumption
TE: Total effect

## References

[1] Anderson AS, Loeser RF (2010). Why is osteoarthritis an age-related disease?. Best Pract Res Clin Rheumatol.

[2] van Dijk GM, Veenhof C, Spreeuwenberg P, Coene N, Burger BJ, van Schaardenburg D (2010). Prognosis of limitations in activities in osteoarthritis of the hip or knee: a 3-year cohort study. Arch Phys Med Rehabil.

[3] World Health Organization. The burden of musculoskeletal conditions at the start of the new millennium: report of a WHO Scientific Group: World Health Organization; 2003. 14679827

[4] Department of Economic and Social Affairs PD. World Population Ageing 2019 Highlights United Nations; 2019.

[5] Ilsan hospital national health insurance service. Inflammation of the joints or cartilage caused by the wear of cartilage is common in the 50s and 60s. 2016.

[6] Donabedian A. Aspects of medical care administration: specifying requirements for health care: Harvard University press; 1973.

[7] Chen J, Hou F (2002). Unmet needs for health care. Health Rep.

[8] Kjeken I, Dagfinrud H, Slatkowsky-Christensen B, Mowinckel P, Uhlig T, Kvien TK (2005). Activity limitations and participation restrictions in women with hand osteoarthritis: patients’ descriptions and associations between dimensions of functioning. Ann Rheum Dis.

[9] McColl MA, Jarzynowska A, Shortt S (2010). Unmet health care needs of people with disabilities: population level evidence. Disabil Soc.

[10] Moran C, Horton T. Total knee replacement: the joint of the decade: a successful operation, for which there’s a large unmet need. British Medical Journal Publishing Group; 2000. 10.1136/bmj.320.7238.820 PMC1127183 10731156

[11] Laufer S (2004). Osteoarthritis therapy—are there still unmet needs?. Rheumatology.

[12] Korea centers for disease control & prevention. The Korea National Health and Nutrition Examination Survey VI(2013-2015). 2015.

[13] Korea centers for disease control & prevention. Kyung Hee University Industry-Academic Collaboration Foundation. Professional Surveyer Education and Quality Control for Osteoarthritis Examination the fifth Korea National Health and Nutrition Examination Survey (KNHANES V-1), 2010. 2010.

[14] Park J-H, Hong J-Y, Han K, Suh S-W, Park S-Y, Yang J-H, et al. Prevalence of symptomatic hip, knee, and spine osteoarthritis nationwide health survey analysis of an elderly Korean population. Medicine. 2017;96(12). 10.1097/MD.0000000000006372 PMC5371462 28328825

[15] Yoshimura N, Muraki S, Oka H, Mabuchi A, En-Yo Y, Yoshida M (2009). Prevalence of knee osteoarthritis, lumbar spondylosis, and osteoporosis in Japanese men and women: the research on osteoarthritis/osteoporosis against disability study. J Bone Miner Metab.

[16] Blagojevic M, Jinks C, Jeffery A, Jordan K (2010). Risk factors for onset of osteoarthritis of the knee in older adults: a systematic review and meta-analysis. Osteoarthr Cartil.

[17] Young-ho J (2012). Limitations in activities and unmet needs health in Korea medical insurance. Korea Inst Health Soc Affairs Issue Focus.

[18] Srikanth VK, Fryer JL, Zhai G, Winzenberg TM, Hosmer D, Jones G (2005). A meta-analysis of sex differences prevalence, incidence and severity of osteoarthritis. Osteoarthr Cartil.

[19] Hwang J (2018). Understanding reasons for unmet health care needs in Korea: what are health policy implications?. BMC Health Serv Res.

[20] Kotlarz H, Gunnarsson CL, Fang H, Rizzo JA (2009). Insurer and out-of-pocket costs of osteoarthritis in the US: evidence from national survey data. Arthritis Rheumatism.

[21] Lee S-Y, Kim C-W, Kang J-H, Seo N-K (2015). Unmet healthcare needs depending on employment status. Health Policy..

[22] Cleveland RJ, Schwartz TA, Prizer LP, Randolph R, Schoster B, Renner JB (2013). Associations of educational attainment, occupation, and community poverty with hip osteoarthritis. Arthritis Care Res.

[23] Felson DT, Lawrence RC, Dieppe PA, Hirsch R, Helmick CG, Jordan JM (2000). Osteoarthritis: new insights. Part 1: the disease and its risk factors. Ann Intern Med.

[24] Han K-T, Park E-C, Kim SJ (2016). Unmet healthcare needs and community health center utilization among the low-income population based on a nationwide community health survey. Health Policy.

[25] van Dijk GM, Veenhof C, Schellevis F, Hulsmans H, Bakker JP, Arwert H (2008). Comorbidity, limitations in activities and pain in patients with osteoarthritis of the hip or knee. BMC Musculoskelet Disord.

[26] Ho SJ. Quality control and assurance of blood pressure measurement - KNHANES 6 (2013). Korea centers for disease control & prevention. Hanyang University Industry-Academic Collaboration Foundation; 2013.

[27] Hawker GA, Gignac MA, Badley E, Davis AM, French MR, Li Y (2011). A longitudinal study to explain the pain-depression link in older adults with osteoarthritis. Arthritis Care Res.

[28] Kim KW, Han JW, Cho HJ, Chang CB, Park JH, Lee JJ (2011). Association between comorbid depression and osteoarthritis symptom severity in patients with knee osteoarthritis. JBJS..

[29] Valderrabano V, Steiger C. Treatment and prevention of osteoarthritis through exercise and sports. J Aging Res. 2011;2011. 10.4061/2011/374653 PMC3004403 21188091

[30] Sugiyama T, Steers WN, Wenger NS, Duru OK, Mangione CM (2015). Effect of a community-based diabetes self-management empowerment program on mental health-related quality of life: a causal mediation analysis from a randomized controlled trial. BMC Health Serv Res.

[31] Kim Y, Kim S, Jeong S, Cho SG, Hwang S (2019). Poor people and poor health: examining the mediating effect of unmet healthcare needs in Korea. J Prevent Med Public Health.

[32] Keele L, Tingley D, Yamamoto T (2015). Identifying mechanisms behind policy interventions via causal mediation analysis. J Policy Anal Manag.

[33] Imai K, Keele L, Tingley D (2010). A general approach to causal mediation analysis. Psychol Methods.

[34] VanderWeele TJ, Vansteelandt S (2010). Odds ratios for mediation analysis for a dichotomous outcome. Am J Epidemiol.

[35] Austin PC (2011). Optimal caliper widths for propensity-score matching when estimating differences in means and differences in proportions in observational studies. Pharm Stat.

[36] Newacheck PW, Stoddard JJ, Hughes DC, Pearl M (1998). Health insurance and access to primary care for children. N Engl J Med.

[37] Glyn-Jones S, Palmer A, Agricola R, Price A, Vincent T, Weinans H (2015). Osteoarthritis. Lancet.

[38] Kim H, Cho S-K, Kim D, Kim D, Jung S-Y, Jang EJ, et al. Impact of osteoarthritis on household catastrophic health expenditures in Korea. J Korean Med Sci. 2018;33(21). 10.3346/jkms.2018.33.e161 PMC5955739 29780297

[39] Suh NK, Kang TW, Heo SI, Lee HJ, Kim DS, Byung-Mook L, et al. Korean Health System Focused on Health Index. National Health Insurance & Korea Institute for Health and Social Affairs; 2016.

[40] Israel S (2016). How social policies can improve financial accessibility of healthcare: a multi-level analysis of unmet medical need in European countries. Int J Equity Health.

