# Supplementary material for: Association between osteoarthritis and unmet medical needs in Korea: limitations in activities as a mediator
Source: BMC Public Health. 2020 Jun 29;20:1026. doi: 10.1186/s12889-020-09140-3 (PMC7325304; doi:10.1186/s12889-020-09140-3)
Supplement: Supplementary file 1 — Additional file 1. Table S1. Sociodemographic characteristics of the population according to severity. Values are presented as number (percentage) and mean ± standard deviation. OA: Osteoarthritis. [file 12889_2020_9140_MOESM1_ESM.docx]

| Additional file 1 Sociodemographic characteristics of the population according to severity* | | | | |
| --- | --- | --- | --- | --- |
|  | **Multi joint OA** | **Single joint OA** | **Non-OA** |  |
|  | **(N = 652)** | **(N = 2,130)** | **(N = 7,347)** | **P value** |
| **Age** | 70.2 ± 7.9 | 66.1 ± 8.6 | 61.7 ± 8.4 | <0.001 |
| **Sex** |  |  |  | <0.001 |
| Men | 96 (14.7) | 646 (30.3) | 3638 (49.5) |  |
| Women | 556 (85.3) | 1484 (69.7) | 3709 (50.5) |  |
| **Marriage status** |  |  |  | <0.001 |
| Cohabitant, spouse | 344 (52.8) | 1539 (72.3) | 6168 (84.0) |  |
| Divorced, separated, single, widowed | 308 (47.2) | 591 (27.7) | 1179 (16.0) |  |
| **Household composition** |  |  |  | <0.001 |
| One-person household | 179 (27.5) | 316 (14.8) | 621 ( 8.5) |  |
| One-generation household | 231 (35.4) | 886 (41.6) | 2702 (36.8) |  |
| Two-generation household | 157 (24.1) | 661 (31.0) | 3233 (44.0) |  |
| Three-generation household or more | 85 (13.0) | 267 (12.5) | 791 (10.8) |  |
| **Household income** |  |  |  | <0.001 |
| Lower | 372 (57.1) | 892 (41.9) | 1718 (23.4) |  |
| Lower middle | 131 (20.1) | 588 (27.6) | 1908 (26.0) |  |
| Upper middle | 82 (12.6) | 347 (16.3) | 1757 (23.9) |  |
| Upper | 67 (10.3) | 303 (14.2) | 1964 (26.7) |  |
| **Residence** |  |  |  | <0.001 |
| Town | 379 (58.1) | 1377 (64.6) | 5694 (77.5) |  |
| Rural | 273 (41.9) | 753 (35.4) | 1653 (22.5) |  |
| **Education level** |  |  |  | <0.001 |
| Elementary school graduate or lower | 540 (82.8) | 1366 (64.1) | 2687 (36.6) |  |
| Middle school graduate | 69 (10.6) | 347 (16.3) | 1350 (18.4) |  |
| High school graduate | 34 ( 5.2) | 310 (14.6) | 2176 (29.6) |  |
| College graduate or higher | 9 ( 1.4) | 107 ( 5.0) | 1134 (15.4) |  |
| **Occupation** |  |  |  | <0.001 |
| White collar and service | 51 ( 7.8) | 243 (11.4) | 1655 (22.5) |  |
| Blue collar and farmer | 190 (29.1) | 744 (34.9) | 2514 (34.2) |  |
| Unemployed | 411 (63.0) | 1143 (53.7) | 3178 (43.3) |  |
| **Type of insurance** |  |  |  | <0.001 |
| Medical aid system | 49 ( 7.5) | 99 ( 4.6) | 183 ( 2.5) |  |
| National health insurance | 603 (92.5) | 2031 (95.4) | 7164 (97.5) |  |
| **Private insurance** |  |  |  | <0.001 |
| No | 433 (66.4) | 1122 (52.7) | 2610 (35.5) |  |
| Yes | 219 (33.6) | 1008 (47.3) | 4737 (64.5) |  |
| **Current status of smoking** |  |  |  | <0.001 |
| No | 599 (91.9) | 1863 (87.5) | 6098 (83.0) |  |
| Yes | 53 ( 8.1) | 267 (12.5) | 1249 (17.0) |  |
| **Monthly status of drinking** |  |  |  | <0.001 |
| No | 474 (72.7) | 1380 (64.8) | 3795 (51.7) |  |
| Yes | 178 (27.3) | 750 (35.2) | 3552 (48.3) |  |
| **Execution of muscle strengthening exercise** |  |  |  | <0.001 |
| No | 607 (93.1) | 1838 (86.3) | 5567 (75.8) |  |
| Yes | 45 ( 6.9) | 292 (13.7) | 1780 (24.2) |  |
| **Depression** |  |  |  | <0.001 |
| No | 618 (94.8) | 2040 (95.8) | 7185 (97.8) |  |
| Yes | 34 ( 5.2) | 90 ( 4.2) | 162 ( 2.2) |  |
| **Obesity** |  |  |  | <0.001 |
| No | 336 (51.5) | 1302 (61.1) | 4925 (67.0) |  |
| Yes | 316 (48.5) | 828 (38.9) | 2422 (33.0) |  |
| **Hypertension** |  |  |  | <0.001 |
| No | 243 (37.3) | 912 (42.8) | 3927 (53.5) |  |
| Yes | 409 (62.7) | 1218 (57.2) | 3420 (46.5) |  |
| **Diabetes** |  |  |  | <0.001 |
| No | 495 (75.9) | 1735 (81.5) | 6119 (83.3) |  |
| Yes | 157 (24.1) | 395 (18.5) | 1228 (16.7) |  |
| **Hyperlipidemia** |  |  |  | <0.001 |
| No | 471 (72.2) | 1601 (75.2) | 5759 (78.4) |  |
| Yes | 181 (27.8) | 529 (24.8) | 1588 (21.6) |  |
| * Values are presented as number (percentage) and mean ± standard deviation. *OA:* steoarthritis; | | | | |
