# Supplementary material for: Association between osteoarthritis and unmet medical needs in Korea: limitations in activities as a mediator
Source: BMC Public Health. 2020 Jun 29;20:1026. doi: 10.1186/s12889-020-09140-3 (PMC7325304; doi:10.1186/s12889-020-09140-3)
Supplement: Supplementary file 2 — Additional file 2. Table S2. Sociodemographic characteristics of the population according to severity by mediators. Values are presented as number (percentage) and mean ± standard deviation. OA: Osteoarthritis. [file 12889_2020_9140_MOESM2_ESM.docx]

| **Additional file 2** **Sociodemographic characteristics of the population according to severity by mediators** | | | | | | | | | |
| --- | --- | --- | --- | --- | --- | --- | --- | --- | --- |
|  | **Multi joint OA (N = 652)** | | | **Single joint OA (N = 2,130)** | | | **Non-OA (N = 7,347)** | | |
| **Limitations in activities** | **Yes** | **No** | **P value** | **Yes** | **No** | **P value** | **Yes** | **No** | **P value** |
|  | (N=268) | (N=384) |  | (N=609) | (N=1521) |  | (N=657) | (N=6690) |  |
| **Age** | 70.9 ± 8.0 | 69.7 ± 7.8 | 0.076 | 67.4 ± 8.4 | 65.6 ± 8.6 | <0.001 | 63.7 ± 8.5 | 61.5 ± 8.4 | <0.001 |
| **Sex** |  |  | 0.071 |  |  | 0.023 |  |  | 0.923 |
| Men | 48 (17.9%) | 48 (12.5%) |  | 207 (34.0%) | 439 (28.9%) |  | 327 (49.8%) | 3311 (49.5%) |  |
| Women | 220 (82.1%) | 336 (87.5%) |  | 402 (66.0%) | 1082 (71.1%) |  | 330 (50.2%) | 3379 (50.5%) |  |
| **Marriage status** |  |  | 0.04 |  |  | 0.554 |  |  | <0.001 |
| Cohabitant, spouse | 128 (47.8%) | 216 (56.2%) |  | 434 (71.3%) | 1105 (72.6%) |  | 508 (77.3%) | 5660 (84.6%) |  |
| Divorced, separated, single, widowed | 140 (52.2%) | 168 (43.8%) |  | 175 (28.7%) | 416 (27.4%) |  | 149 (22.7%) | 1030 (15.4%) |  |
| **Household composition** |  |  | 0.005 |  |  | 0.225 |  |  | <0.001 |
| One-person household | 93 (34.7%) | 86 (22.4%) |  | 97 (15.9%) | 219 (14.4%) |  | 82 (12.5%) | 539 (8.1%) |  |
| One-generation household | 86 (32.1%) | 145 (37.8%) |  | 268 (44.0%) | 618 (40.6%) |  | 273 (41.6%) | 2429 (36.3%) |  |
| Two-generation household | 61 (22.8%) | 96 (25.0%) |  | 172 (28.2%) | 489 (32.1%) |  | 238 (36.2%) | 2995 (44.8%) |  |
| Three-generation household or more | 28 (10.4%) | 57 (14.8%) |  | 72 (11.8%) | 195 (12.8%) |  | 64 (9.7%) | 727 (10.9%) |  |
| **Household income** |  |  | 0.163 |  |  | <0.001 |  |  | <0.001 |
| Lower | 164 (61.2%) | 208 (54.2%) |  | 313 (51.4%) | 579 (38.1%) |  | 247 (37.6%) | 1471 (22.0%) |  |
| Lower middle | 52 (19.4%) | 79 (20.6%) |  | 167 (27.4%) | 421 (27.7%) |  | 176 (26.8%) | 1732 (25.9%) |  |
| Upper middle | 32 (11.9%) | 50 (13.0%) |  | 75 (12.3%) | 272 (17.9%) |  | 135 (20.5%) | 1622 (24.2%) |  |
| Upper | 20 (7.5%) | 47 (12.2%) |  | 54 (8.9%) | 249 (16.4%) |  | 99 (15.1%) | 1865 (27.9%) |  |
| **Residence** |  |  | 0.447 |  |  | 0.001 |  |  | 0.034 |
| Town | 161 (60.1%) | 218 (56.8%) |  | 360 (59.1%) | 1017 (66.9%) |  | 487 (74.1%) | 5207 (77.8%) |  |
| Rural | 107 (39.9%) | 166 (43.2%) |  | 249 (40.9%) | 504 (33.1%) |  | 170 (25.9%) | 1483 (22.2%) |  |
| **Education level** |  |  | 0.402 |  |  | <0.001 |  |  | <0.001 |
| Elementary school graduate or lower | 226 (84.3%) | 314 (81.8%) |  | 419 (68.8%) | 947 (62.3%) |  | 319 (48.6%) | 2368 (35.4%) |  |
| Middle school graduate | 27 (10.1%) | 42 (10.9%) |  | 106 (17.4%) | 241 (15.8%) |  | 118 (18.0%) | 1232 (18.4%) |  |
| High school graduate | 10 (3.7%) | 24 (6.2%) |  | 66 (10.8%) | 244 (16.0%) |  | 151 (23.0%) | 2025 (30.3%) |  |
| College graduate or higher | 5 (1.9%) | 4 (1.0%) |  | 18 (3.0%) | 89 (5.9%) |  | 69 (10.5%) | 1065 (15.9%) |  |
| **Occupation** |  |  | <0.001 |  |  | <0.001 |  |  | <0.001 |
| White collar and service | 13 (4.9%) | 38 (9.9%) |  | 36 (5.9%) | 207 (13.6%) |  | 78 (11.9%) | 1577 (23.6%) |  |
| Blue collar and farmer | 57 (21.3%) | 133 (34.6%) |  | 182 (29.9%) | 562 (36.9%) |  | 172 (26.2%) | 2342 (35.0%) |  |
| Unemployed | 198 (73.9%) | 213 (55.5%) |  | 391 (64.2%) | 752 (49.4%) |  | 407 (61.9%) | 2771 (41.4%) |  |
| **Type of insurance** |  |  | 0.012 |  |  | <0.001 |  |  | <0.001 |
| Medical aid system | 29 (10.8%) | 20 (5.2%) |  | 49 (8.0%) | 50 (3.3%) |  | 61 (9.3%) | 122 (1.8%) |  |
| National health insurance | 239 (89.2%) | 364 (94.8%) |  | 560 (92.0%) | 1471 (96.7%) |  | 596 (90.7%) | 6568 (98.2%) |  |
| **Private insurance** |  |  | 0.798 |  |  | <0.001 |  |  | <0.001 |
| No | 180 (67.2%) | 253 (65.9%) |  | 368 (60.4%) | 754 (49.6%) |  | 345 (52.5%) | 2265 (33.9%) |  |
| Yes | 88 (32.8%) | 131 (34.1%) |  | 241 (39.6%) | 767 (50.4%) |  | 312 (47.5%) | 4425 (66.1%) |  |
| **Current status of smoking** |  |  | 0.279 |  |  | 0.106 |  |  | 0.223 |
| No | 242 (90.3%) | 357 (93.0%) |  | 521 (85.6%) | 1342 (88.2%) |  | 557 (84.8%) | 5541 (82.8%) |  |
| Yes | 26 (9.7%) | 27 (7.0%) |  | 88 (14.4%) | 179 (11.8%) |  | 100 (15.2%) | 1149 (17.2%) |  |
| **Monthly status of drinking** |  |  | 0.234 |  |  | 0.11 |  |  | <0.001 |
| No | 202 (75.4%) | 272 (70.8%) |  | 411 (67.5%) | 969 (63.7%) |  | 409 (62.3%) | 3386 (50.6%) |  |
| Yes | 66 (24.6%) | 112 (29.2%) |  | 198 (32.5%) | 552 (36.3%) |  | 248 (37.7%) | 3304 (49.4%) |  |
| **Execution of muscle strengthening exercise** |  |  | 1 |  |  | 0.026 |  |  | 0.024 |
| No | 250 (93.3%) | 357 (93.0%) |  | 542 (89.0%) | 1296 (85.2%) |  | 522 (79.5%) | 5045 (75.4%) |  |
| Yes | 18 (6.7%) | 27 (7.0%) |  | 67 (11.0%) | 225 (14.8%) |  | 135 (20.5%) | 1645 (24.6%) |  |
| **Depression** |  |  | 0.019 |  |  | <0.001 |  |  | <0.001 |
| No | 247 (92.2%) | 371 (96.6%) |  | 560 (92.0%) | 1480 (97.3%) |  | 610 (92.8%) | 6575 (98.3%) |  |
| Yes | 21 (7.8%) | 13 (3.4%) |  | 49 (8.0%) | 41 (2.7%) |  | 47 (7.2%) | 115 (1.7%) |  |
| **Obesity** |  |  | 0.678 |  |  | 0.058 |  |  | 0.607 |
| No | 135 (50.4%) | 201 (52.3%) |  | 392 (64.4%) | 910 (59.8%) |  | 434 (66.1%) | 4491 (67.1%) |  |
| Yes | 133 (49.6%) | 183 (47.7%) |  | 217 (35.6%) | 611 (40.2%) |  | 223 (33.9%) | 2199 (32.9%) |  |
| **Hypertension** |  |  | 0.79 |  |  | 0.019 |  |  | 0.052 |
| No | 102 (38.1%) | 141 (36.7%) |  | 236 (38.8%) | 676 (44.4%) |  | 327 (49.8%) | 3600 (53.8%) |  |
| Yes | 166 (61.9%) | 243 (63.3%) |  | 373 (61.2%) | 845 (55.6%) |  | 330 (50.2%) | 3090 (46.2%) |  |
| **Diabetes** |  |  | 0.995 |  |  | 0.121 |  |  | <0.001 |
| No | 204 (76.1%) | 291 (75.8%) |  | 483 (79.3%) | 1252 (82.3%) |  | 501 (76.3%) | 5618 (84.0%) |  |
| Yes | 64 (23.9%) | 93 (24.2%) |  | 126 (20.7%) | 269 (17.7%) |  | 156 (23.7%) | 1072 (16.0%) |  |
| **Hyperlipidemia** |  |  | 0.845 |  |  | 0.56 |  |  | 0.003 |
| No | 192 (71.6%) | 279 (72.7%) |  | 452 (74.2%) | 1149 (75.5%) |  | 485 (73.8%) | 5274 (78.8%) |  |
| Yes | 76 (28.4%) | 105 (27.3%) |  | 157 (25.8%) | 372 (24.5%) |  | 172 (26.2%) | 1416 (21.2%) |  |
