# Supplementary figures and images for: Association between osteoarthritis and unmet medical needs in Korea: limitations in activities as a mediator
Source: BMC Public Health. 2020 Jun 29;20:1026. doi: 10.1186/s12889-020-09140-3 (PMC7325304; doi:10.1186/s12889-020-09140-3)

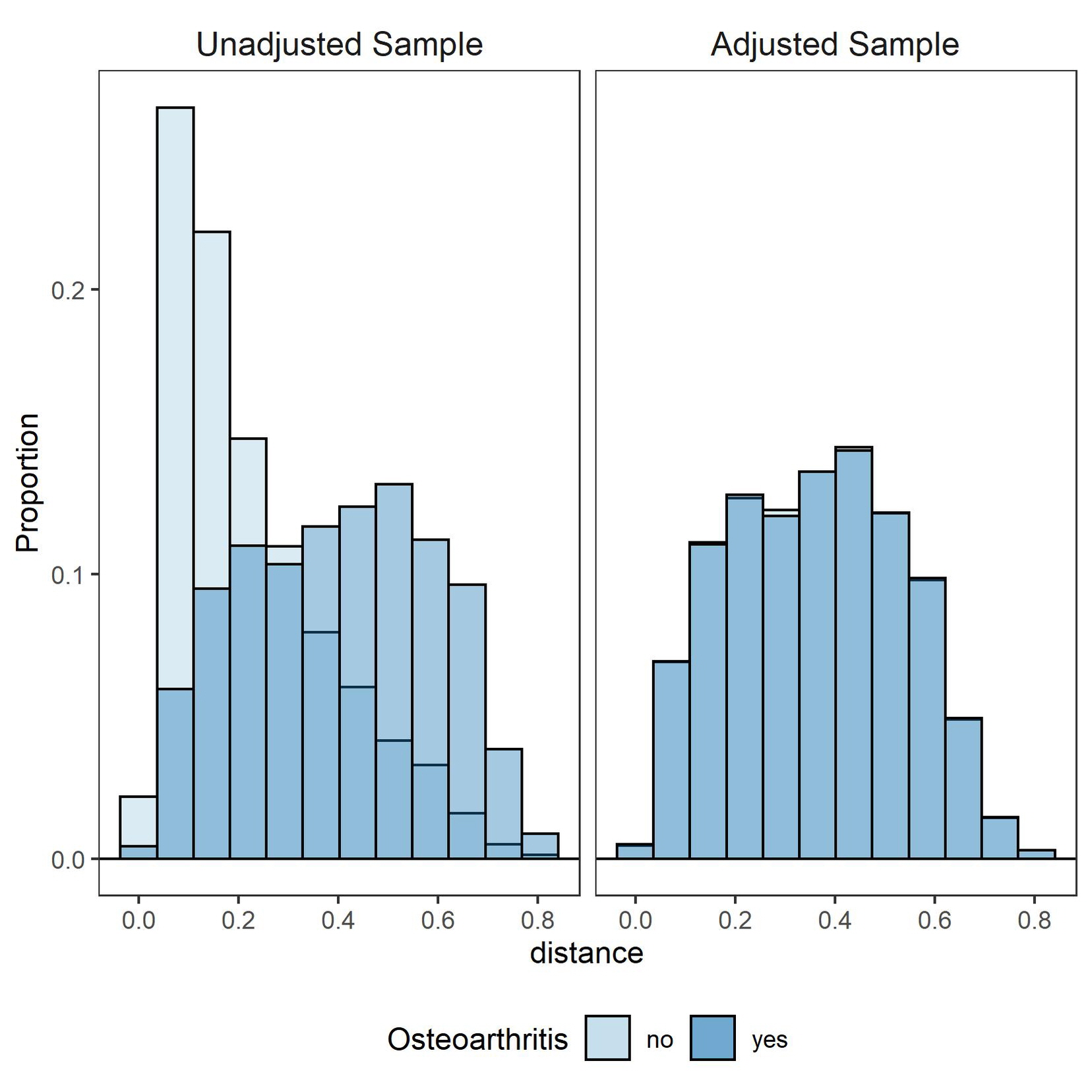

Supplement: Supplementary file 3 — Additional file 3. Figure S1. Propensity score distribution in the overall and matched study populations. 0.01 caliper was used for matching. [file 12889_2020_9140_MOESM3_ESM.jpg]
