# Supplementary material for: Association between osteoarthritis and unmet medical needs in Korea: limitations in activities as a mediator
Source: BMC Public Health. 2020 Jun 29;20:1026. doi: 10.1186/s12889-020-09140-3 (PMC7325304; doi:10.1186/s12889-020-09140-3)
Supplement: Supplementary file 4 — Additional file 4. Table S3. Effects on unmet needs are presented as odds ratio (95% confidence interval). Propensity score matching using nearest method with 0.01 caliper was used. TE total effects, NDE natural direct effects, NIE natural indirect effects. [file 12889_2020_9140_MOESM4_ESM.docx]

| **Mediator variables** | **Total Effect** | **Natural Direct Effect** | **Natural Indirect Effect** | **% of total**  **effect mediated** | **Interaction(P value)** |
| --- | --- | --- | --- | --- | --- |
| All cause of unmet need | 1.65 (1.53 - 1.79) | 1.55 (1.42 - 1.69) | 1.07 (1.05 - 1.09) | 12.8 | 0.014 |
| Availability | 2.13 (1.66 - 2.71) | 2.11 (1.64 - 2.75) | 1.01 (0.96 - 1.06) | 1.5 | 0.09 |
| Accessibility | 2.32 (1.93 - 2.75) | 2.02 (1.67 - 2.38) | 1.15 (1.10 - 1.21) | 17.4 | 0.07 |
| financial | 2.41 (1.99 - 2.89) | 2.09 (1.73 - 2.51) | 1.15 (1.10 - 1.21) | 16.7 | 0.08 |
| transportation | 2.60 (1.59 - 4.19) | 2.12 (1.25 - 3.42) | 1.23 (1.08 - 1.42) | 22.8 | 0.50 |
| Acceptability | 1.40 (1.12 - 1.72) | 1.40 (1.11 - 1.71) | 1.00 (0.96 - 1.06) | 0.7 | 0.07 |
